# Role of Non-Coding Variants in Brugada Syndrome

**DOI:** 10.3390/ijms21228556

**Published:** 2020-11-13

**Authors:** Adrian Pérez-Agustín, Mel·lina Pinsach-Abuin, Sara Pagans

**Affiliations:** 1 Department of Medical Sciences, School of Medicine, University of Girona, 17003 Girona, Spain; adriaperez@gencardio.com; 2 Biomedical Research Institute of Girona, 17190 Salt, Spain; mpinsach@gencardio.com

**Keywords:** Brugada syndrome, arrhythmia, sudden cardiac death, non-coding variants, gene regulation, *cis*-regulatory elements, non-coding RNAs, *SCN5A*, *SCN10A*

## Abstract

Brugada syndrome (BrS) is an inherited electrical heart disease associated with a high risk of sudden cardiac death (SCD). The genetic characterization of BrS has always been challenging. Although several cardiac ion channel genes have been associated with BrS, *SCN5A* is the only gene that presents definitive evidence for causality to be used for clinical diagnosis of BrS. However, more than 65% of diagnosed cases cannot be explained by variants in *SCN5A* or other genes. Therefore, in an important number of BrS cases, the underlying mechanisms are still elusive. Common variants, mostly located in non-coding regions, have emerged as potential modulators of the disease by affecting different regulatory mechanisms, including transcription factors (TFs), three-dimensional organization of the genome, or non-coding RNAs (ncRNAs). These common variants have been hypothesized to modulate the interindividual susceptibility of the disease, which could explain incomplete penetrance of BrS observed within families. Altogether, the study of both common and rare variants in parallel is becoming increasingly important to better understand the genetic basis underlying BrS. In this review, we aim to describe the challenges of studying non-coding variants associated with disease, re-examine the studies that have linked non-coding variants with BrS, and provide further evidence for the relevance of regulatory elements in understanding this cardiac disorder.

## 1. Introduction

Brugada syndrome (BrS) was first described in 1992 as a rare inherited cardiac disease. It is characterized by a distinctive electrocardiographic pattern with a coved-type ST-segment elevation in the right precordial leads [1]. This disorder is associated with a high risk of sudden cardiac death (SCD) among the population, and has been found to be responsible for 15–20% of supraventricular arrhythmias and atrial fibrillation (AF) cases in structural normal hearts [2].

To date, the best-known cause of BrS is the presence of variants at the coding regions of the *SCN5A* gene, accounting for approximately 20–25% of BrS cases [3]. *SCN5A* encodes the α-subunit of the voltage-gated cardiac sodium channel (Na_V_1.5), which is responsible for the rapid influx of sodium ions that initiates the propagation of the action potential in cardiac muscle cells [4]. These variants in *SCN5A* include single nucleotide variants (SNVs), as well as small insertions and deletions (Indels), and have been associated with Na_V_1.5 loss of function, either affecting its channel activity or reducing the number of channels at the plasma membrane [3]. Other genes, such as *SCN1B*, *SCN2B*, *SCN3B*, *CACNA1C*, *CACNB2*, or *CACNA2D1*, have also been associated with the disease with a lower incidence, representing 5–10% of BrS cases [5,6,7]. However, their relevance is still questioned because of their lack of definitive evidence for causality [8]. Therefore, in more than 65% of BrS diagnosed patients, the etiology of the disease is still unknown.

BrS has traditionally been considered a monogenic disease with an autosomal dominant mode of transmission [9]. However, in recent years, several observations have provided evidence to suggest that BrS has a more complex inheritance and a heterogeneous genetic basis [10,11,12]. Furthermore, it has been observed that BrS-associated variants show low penetrance [10,13]. For instance, studies conducted in families carrying *SCN5A* pathogenic variants have estimated a BrS penetrance of 12.5–50%, with an average of 16% [9]. Additionally, it is well-established that age, gender, environmental factors, and comorbidities contribute to the BrS phenotype [14]. As new genetic studies appear, it is becoming more evident that both coding and non-coding regions play a fundamental role in the pathophysiology of inherited human diseases, including BrS [10]. The challenges surrounding the complex genetics of BrS, and the evidence suggesting that this disorder may not be caused by single genetic variants, have also been recently discussed by Monasky and colleagues [15]. 

Historically, most genetic studies have been focused on studying variants in coding regions. However, during the last ten years, genome-wide association studies (GWAS) have uncovered that most disease-associated variants lie within non-coding regions, especially at *cis*-regulatory regions [16]. This has led to an increasing interest in examining the role of regulatory variants in inherited diseases. In this regard, a recent analysis of 150 million SNVs in 10,000 human genomes revealed that genetic variation is unevenly distributed across the human genome, and that promoters, transcription factor binding sites (TFBSs), open-chromatin regions, and CCCTC-binding factor (CTCF) sites, in this order, accumulate the largest number of SNVs in regulatory elements across the human genome [17]. Similarly, it has been proposed that the presence of variants at *cis*-regulatory elements (CREs) and non-coding RNAs (ncRNAs) involved in modulating the expression levels of cardiac ion channels could be a mechanism underlying the BrS phenotype [12,18].

From GWAS and other genetic studies, we have learnt that there is often a correlation between the severity and frequency of variants, where variants with a major impact are rare (minor allele frequency (MAF) < 1%) and those with limited impact are common (MAF ≥ 5%) [19]. As a result, common variants could be exacerbating or attenuating the disease manifestation alongside a primary genetic defect, which is usually described in a coding region, but these variants can also be responsible for the disease phenotype in the absence of coding variants [20]. A further source of complexity is that a common variant in one particular population can be rare in another one [21]. Therefore, it has been suggested that, in some cases, the disease phenotype might be modulated through an abnormal combination of common variants [22,23]. This has led to an increasing interest in common non-coding variants as potential modulators of the BrS phenotype by fine-tuning the effect of coding variants. Indeed, similar synergistic effects with rare variants have already been described for common variants in the *SCN5A* coding region [24].

## 2. The Challenges Surrounding Non-Coding Variants

### 2.1. Functional Effects of Non-Coding Variants

The functional effect of variants in coding regions is easy to predict, since the genetic code is already known and there is an important amount of available exome data, as well as a good knowledge of Mendelian disorders [25]. However, the effect of variants in non-coding regions is difficult to assess. These non-coding variants can be found within CREs, such as enhancers, promoters, and insulators, as well as regions that encode ncRNAs. Importantly, all these elements precisely coordinate gene expression, both in a spatial and temporal way [26]. 

Promoters, enhancers, and insulators harbour TFBSs that regulate their activity [27]. An enhancer is a DNA element recognized by specific TFs that increase transcription of its regulated genes. Enhancers can be located either upstream or downstream from the transcription start site (TSS), and up to 1 Mb away from their target gene [28]. In contrast, promoters are DNA elements recognized by general TFs and the RNA polymerase, and are located near the TSS [29]. On a more global scale, insulators control the accessibility of regulatory elements between well-defined genomic territories, known as topological associating domains (TADs), which are hubs of chromatin interactions that establish the limits of action of regulatory regions in the human genome [30,31].

**Figure 1:**
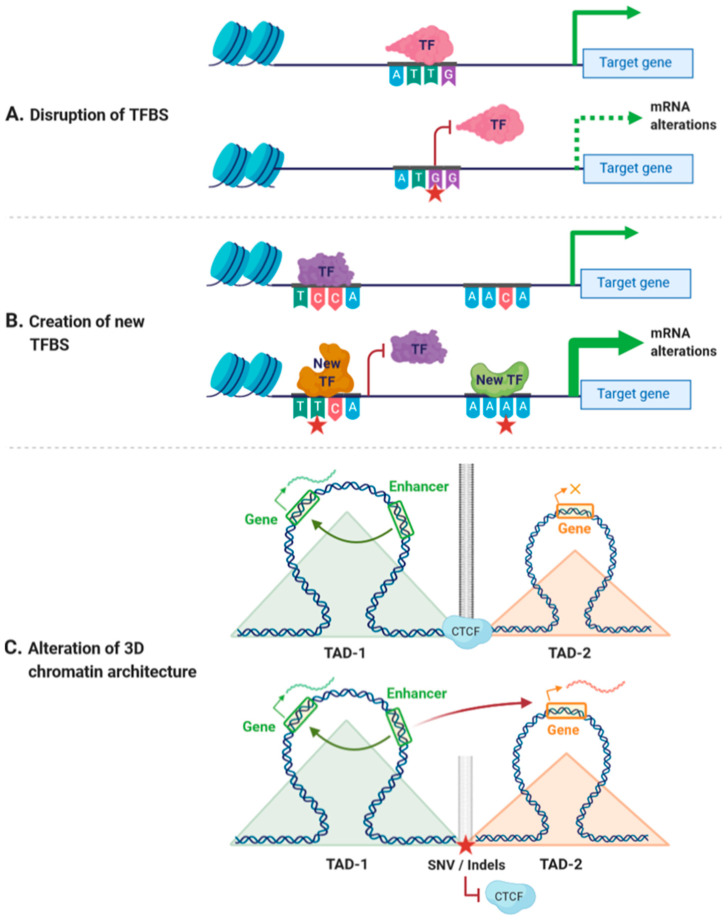
Effects of *cis*-regulatory variants. (**A**) Variants within *cis*-regulatory regions can disrupt transcription factor binding sites (TFBSs), leading to decreased mRNA expression. (**B**) Variants within *cis*-regulatory regions can create new TFBSs and generate new regulatory pathways. (**C**) Variants affecting the binding of CCCTC-binding factor (CTCF) can disrupt insulator activity between two topological associating domains (TADs), generating new enhancer–promoter interactions that may lead to altered expression of genes within these TADs. Red star indicates the position of a new single nucleotide variant (SNV) or an insertion/deletion (Indel). Orange × indicates inhibition of gene expression.

*Cis*-regulatory variants could have different outcomes depending on the nucleotide change and the position of the variant. For instance, a *cis*-regulatory, non-coding variant could disrupt the binding of TFs to CREs, changing the accessibility of DNA to TFs or altering the spatial organization of the genome [32,33,34] (Figure 1A). A unique regulatory variant could also create a new TFBS, thereby altering the existing regulatory network and generating alternative regulatory pathways [35] (Figure 1B). In addition, non-coding variants affecting the accessibility of DNA have been linked with alterations in the binding of chromatin-binding proteins, which may result in alterations in gene expression [36,37]. Similarly, variants within CTCF-binding sites may affect the spatial organization of the genome, leading to alterations of the transcriptional networks [38,39] (Figure 1C). As an example, variants at CTCF cohesin binding sites have been associated with colorectal cancer and with T-cell acute lymphoblastic leukemia [40,41].

Despite the previous examples, in most cases, alterations in TF binding (either due to variants on the TFBS or the DNA-binding domain of the TF) do not have huge effects on transcription levels, and for this reason they are thought to act as genetic modifiers [42]. TFBS contain nucleotides with different relevance for the binding of TFs. The more conserved nucleotides, also referred to as core nucleotides, are essential for TF binding. Therefore, genetic variants affecting core nucleotides will have a higher impact on TF binding. In contrast, the less conserved nucleotides are thought to confer some tolerance to genetic variation, which might explain why many changes in TFBS do not result in measurable alterations in mRNA levels [43]. In addition, low effects of *cis*-regulatory variants, especially in enhancer elements, could also be explained by the concept of regulatory redundancy [44]. In a recent report, Osterwalder and colleagues observed that deletion of a single enhancer in genes surrounded by multiple enhancers did not result in phenotypic changes, whereas the deletion of more than one resulted in measurable alterations. These findings suggest that enhancers present a robustness to genetic variation when acting together, and that their contribution to gene regulation is more likely redundant rather than additive [45]. In addition, other studies have recently proposed that the regulatory outcome not only depends on the affinity of the TF towards a DNA sequence, but also on a balance between TF affinity and regulatory syntax (order, spacing, and orientation of binding sites), and that an optimal syntax can compensate a weak TFBS, and vice versa [46]. Altogether, the mechanisms of TF binding and promoter/enhancer function reduce the likelihood of large effects produced by a single variant. This would be consistent with multiple studies suggesting that common regulatory variants should be studied in combination and not isolated [15,22].

As mentioned above, non-coding variants can also be found in ncRNAs. ncRNAs are largely classified into microRNAs (miRNAs) and long non-coding RNAs (lncRNAs), although other types of ncRNAs have also been described, including bi-functional coding and non-coding RNAs (cncRNAs) or circular RNAs (circRNAs) [47]. ncRNAs have been shown to have important roles in modulating gene expression at either the mRNA or protein level. They participate in several key functions related to chromatin structure, as well as transcription and post-transcriptional processes [48]. Notably, it is estimated that all known ncRNAs would target more than 60% of the coding genes [49]. 

**Figure 2:**
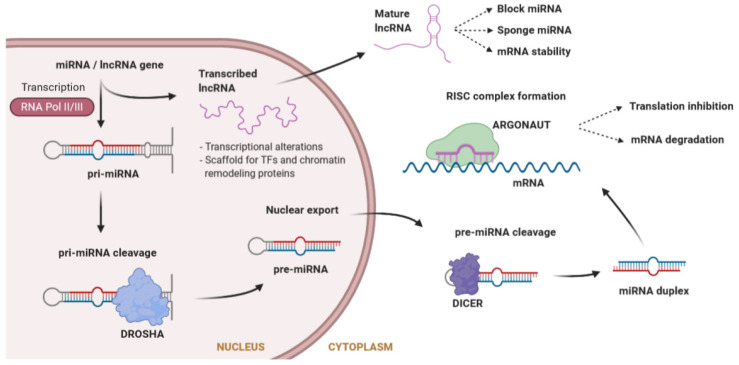
Synthesis of microRNAs (miRNAs) and long non-coding RNAs (lncRNAs), as well as mechanisms of action. miRNA biogenesis consists of a series of steps where a premature miRNA is cleaved until it reaches the mature form outside the nucleus. Both miRNAs and lncRNAs can interfere with gene regulation through different mechanisms. lncRNAs also interfere in the function of other miRNAs.

MicroRNAs (miRNAs) are a large class of evolutionarily conserved ncRNAs, 20 to 26 nucleotides in length, that are expressed in a tissue-specific manner. miRNAs post-transcriptionally regulate the flow of genetic information by inhibiting mRNA translation or promoting mRNA degradation [50]. Typically, miRNAs interact with the 3′ untranslated region (UTR) of specific mRNAs, giving rise to the degradation or stabilization of the target sequence [51]. Large molecules of pri-miRNA are cleaved by Drosha into a pre-miRNA, which is further processed in the cytosol by Dicer to obtain the mature miRNA. Mature miRNAs will load into the RNA-induced silencing complex (RISC) to degrade the target mRNA or inhibit its translation [52] (Figure 2). During recent years, it has become evident that miRNAs play central roles in many fundamental biological processes, and that aberrant expression of miRNAs is causally related to a variety of disease states like cancer, diabetes, or cardiovascular diseases [53,54,55]. miRNAs have been shown to play an essential role in cardiac physiology and pathophysiology [56]. In the cardiac conduction system, specific miRNAs targeting cardiac ion channels have been identified [57], thereby suggesting that variants affecting the function of these miRNAs may have an important impact in modulating the phenotype of inherited cardiac arrhythmias.

lncRNAs are non-protein-coding transcripts longer than 200 nucleotides, involved in a large number of diverse functions due to their ability to fold into different structures [58]. The mechanism of action of lncRNAs is different depending on whether they are in the nucleus or in the cytoplasm (Figure 2). Inside the nucleus, lncRNAs can influence transcription by repressing or activating genes, acting as a scaffold both for TFs, to modulate transcription, and for chromatin remodeling proteins, to modify chromatin accessibility [59]. At a post-transcriptional level, in the cytoplasm, lncRNAs can change the stability of mRNAs, but they can also affect miRNAs by acting as a miRNA sponge (avoiding the repression of the miRNA target) or directly blocking miRNAs. Furthermore, they can bind to ribosomes interfering with the translation of mRNAs [59]. 

During the recent years, an increasing number of lncRNAs have been linked to cardiac disorders. For instance, the so-called Braveheart lncRNA has been associated with cardiac development and disease [60,61], while the lncRNA Fendrr has been shown to be a key factor during cardiac morphogenesis [62,63]. Both Braveheart and Fendrr have been suggested to interact with chromatin regulatory proteins, such as the Polycomb repressive complex 2 (PRC2) [64]. Other studies have reported that the levels of circulating cardiac lncRNAs are altered in patients with cardiomyopathy or heart failure [65,66,67], and several lncRNAs have been recently linked with different heart diseases. For instance, lncRNAs ANRIL and MIAT are considered risk factors associated with coronary disease and myocardial infarction, respectively [68,69].

### 2.2. Deciphering the Funtional Outcome of Non-Coding Variants

In contrast to the study of coding variants, in which the analyses are focused on examining the effect of the variant in protein function, the interpretation of the functional impact of regulatory variants still remains a challenge. Although recent efforts to characterize non-coding sequences have identified many regulatory elements and clarified general aspects of gene regulation, a substantial gap remains between the identification of regulatory elements and a detailed understanding of their function [70]. 

Several computational algorithms have been developed to predict regulatory function and prioritize those non-coding variants that are more likely to have a functional impact [71,72], thereby minimizing the number of variants to be studied in functional assays [73]. The combined annotation-dependent depletion (CADD) score and context-dependent tolerance score (CDTS) are two examples of these computational tools. CADD score is the result of integrating diverse functional annotations into a score of deleteriousness, which strongly correlates with molecular functionality and variant effects [74]. In contrast, CDTS measures how tolerant genomic regions are to genetic variation in the context of surrounding sequences. CDTS is organized into percentiles, with regions found in the first percentile being the least tolerant to genetic variation. Those variants found in regions less tolerant to variation are presumed to have the highest functional impact [75]. Similarly, several algorithms have also been developed to assess the effect of variants in miRNAs. For example, the Vienna RNAfold algorithm predicts the effect of variants on the secondary structure and expression of the miRNA [76], calculating the energy changes of the thermodynamic ensemble of the secondary structure of miRNAs. Altogether, these computational algorithms represent useful pre-screening tools before performing functional assays. However, each algorithm focuses on different aspects of genome biology, which can result in heterogeneous predictions of variant pathogenicity. For this reason, it is recommended to use more than one predictor to select putative candidate variants. 

Classically, the effects of *cis*-regulatory variants on gene expression have been studied one-by-one in reporter assays. These reporter assays are based on a plasmid expressing the luciferase gene under the control of a DNA regulatory region containing the variant of interest. After transfecting cells with the plasmid, luciferase activity is measured in order to assess the impact of the variant over cell function [77]. Although this method is still the gold standard for enhancer/promoter assays, only a limited number of candidate regulatory elements can be tested. In addition, it has to be taken into account that, in these assays, the activity of CREs is analyzed within a plasmid, and therefore, the influences of the three-dimensional (3D) chromatin structure (chromatin territories and boundaries) are missing. In the recent years, thanks to the advances in DNA sequencing, some of these limitations have been overcome by the development of a number of powerful, high-throughput assays for large-scale testing of enhancer activity, known as massive parallel reporter assays (MPRAs). These high-throughput reporter screenings, such as CRE analysis by sequencing (CRE-seq) or self-transcribing active regulatory region sequencing (STARR-seq), allow us to massively measure the activity of regulatory elements in a direct, quantitative, and genome-wide manner [78,79]. 

Since its discovery in 2007, the use of human-induced pluripotent stem cells (hiPSCs) has revolutionized the study of human diseases, as they have the potential to be differentiated into any human cell type [80]. hiPSCs are a versatile tool for exploring molecular and cellular phenotypes in wild-type and patient-derived cells. Cardiomyocytes from patient-derived hiPSCs (hiPSC-CMs) have been used as in vitro models for various inherited cardiac diseases, such as long QT syndrome (LQTS), BrS, or congenital heart disease [81]. However, hiPSC-CMs present several limitations. Differentiation protocols still result in immature cardiomyocytes with incomplete cardiac cell structure. Also, it may be difficult to compare results obtained from hiPSC-CMs, due to the variability produced by either reprogramming protocols used to obtain patient-derived hiPSCs, or the inherent patient-specific genetic background. In this regard, to overcome the effects of genetic background, cells derived from the same patient—therefore showing identical genetic background (i.e., isogenic cells)—are being used. Variants of interest are introduced into these cells by Clustered Regularly Interspaced Short Palindromic Repeats (CRISPR)–Cas9 genome editing approaches, generating modified hiPSCs whose genomes only differ in the variants introduced [82]. 

## 3. Non-Coding Variants Associated with Cardiac Disorders and ECG Traits

GWAS have uncovered an important number of non-coding variants associated with cardiovascular diseases or traits. In fact, the GWAS catalog (https://www.ebi.ac.uk/gwas/) includes more than 50 GWAS publications for almost 300 specific cardiovascular traits [83]. These studies have also identified many loci specifically associated with arrhythmias and electrocardiogram (ECG)-related traits such as the QT, PR, or QRS interval durations [84,85,86,87,88,89,90,91].

The QT interval is the time between ventricular depolarization and repolarization, and is a heritable trait with predisposition to arrhythmias and SCD when its duration is altered. A prolongation of the QT interval has been associated with a higher mortality in patients with cardiovascular disease, but also in healthy populations [92]. An example of a non-coding variant associated with QT interval duration and cardiac repolarization, rs7539120, is located in an enhancer modulating the expression of *NOS1AP* [93,94]. Interestingly, rs7539120 overlaps a DNase I hypersensitivity site, and is associated with an overexpression of *NOS1AP* [93]. A GWAS conducted by Arking and colleagues also identified common variants in linkage disequilibrium (LD) associated with cardiac repolarization at the 5′ upstream region of *NOS1AP* gene [89]. These variants were shown to be strongly linked with the modulation of the QT interval in the general population, pointing to a previously unknown role of the nitric oxide synthase pathway in the heart function, later confirmed by other authors [95]. In addition, these common variants have also been shown to modulate the QT interval and risk of arrhythmia in LQTS patients [96,97]. Similarly, intronic and UTR common variants at the *KCNQ1*, *KCNE1*, *KCNH2*, and *SCN5A* genes have been associated with QT interval and myocardial repolarization [91,98]. 

PR interval and QRS duration are ECG parameters used to evaluate the risk of arrhythmia [90,99]. The PR interval measures the time an electrical impulse takes to travel from the atria to the ventricles (from the sinus node to the Purkinje fibers), and the QRS duration records the depolarization of the ventricles [88]. A prolongation of the PR interval has been associated with a higher risk of AF and mortality [100], while alterations in the QRS duration have been associated with a decrease in life expectancy [101]. In addition to coding variants, common non-coding variants in *CDKN1A*, *TBX5*, and *DKK1* have been associated with alterations in QRS duration in GWAS [88,102]. For instance, a non-coding SNV located at the 3′ UTR at the *SHOX2* gene, encoding a TF with a key role in the development of the sinoatrial node, has been associated with longer PR intervals [103]. This variant creates a novel binding site for the hsa-miR-92b-5p, reducing the expression of *SHOX2*, which promotes a proarrhythmogenic condition that could lead to AF [103]. Similarly, in a recent report, Vincentz and colleagues showed that rs10054375, located in a tissue-specific enhancer of Hand1, leads to severe abnormalities in the ventricular conduction system. Their studies demonstrated that this variant affects the binding site for GATA4, a master regulator of cardiac regulatory networks [104]. However, the region containing the *SCN5A* and *SCN10A* genes represents a major locus that consistently accumulates GWAS hits associated with the PR interval and QRS duration (see Section 5) [92].

In addition to arrhythmias, non-coding variants have also been associated with the pathogenesis of cardiomyopathies. For example, intronic variants in dystrophin, plakophilin, or *MYBPC3* genes have been shown to disrupt alternative splicing and generate truncated proteins, leading to hypertrophic cardiomyopathy, dilated cardiomyopathy, and arrhythmogenic right ventricular cardiomyopathy [105]. Furthermore, miR-350, miR-206, miR-195, and miR-208 have been shown to be abnormally overexpressed in cardiac hypertrophy [106]. In contrast, overexpression of the lncRNA Mhrt has been associated with a protective effect in cardiac hypertrophy [107].

## 4. Role of *SCN5A* Protein-Coding Variants in Brugada Syndrome 

To date, more than 350 rare variants associated with BrS have been described within the *SCN5A* coding regions [108,109], representing 20–25% of clinically diagnosed BrS cases [110]. These variants are scattered along the Na_V_1.5 protein, which consists of four homologous domains joined by so-called linkers, where each domain contains six transmembrane helices linked by intracellular or extracellular loops. Functional studies performed in in vitro expression systems have demonstrated that most of these variants lead to a reduction of the sodium current, either by affecting the functionality of the protein or the amount of channel in the cell membrane [3,111]. However, the exact mechanism that links the reduction of the sodium current with the BrS ECG pattern is still under discussion [112]. In addition to *SCN5A*, variants in the coding regions of other ion channel genes, such as sodium channel regulatory beta subunits (*SCN1B*, *SCN2B*, and *SCN3B*) and calcium channels (*CACNA1C*, *CACNA2D1*, and *CACNB2*) have also been described, and account for 5–10% of BrS cases. Variants in other genes have also been associated with the disease, although they represent a much lower incidence of BrS patients [5,113]. Although those genes are routinely tested for in BrS patients, a recent study by Hossein and colleagues showed that *SCN5A* is the only gene with definitive evidence to be used in clinical diagnosis [8].

A major challenge related to BrS genetics is the lack of correlation between genotype and phenotype. Only 20–25% of the BrS patients carry a *SCN5A* variant, and in those cases, there is a highly variable expressivity among the carriers, hindering the interpretation and relevance of variants [13]. Moreover, *SCN5A* variants have also been shown to have pleiotropic effects (i.e., they can be associated with different phenotypes) [20], as they have been associated with different cardiac arrhythmias, such as LQTS type 3, dilated cardiomyopathy, AF, and other conduction defects [114]. Some studies claim that the absence of coding *SCN5A* variants in BrS patients would be a signal of a benign BrS phenotype, and that these patients should not be catalogued as real BrS patients [115]. However, as mentioned above, several authors agree with the notion that the BrS phenotype would be determined by other factors beyond coding variants, including common variants found in non-coding regions from several loci, as well as environmental factors [15]. 

## 5. Role of Non-Coding Variants in Brugada Syndrome

### 5.1. The SCN5A Promoter

During the last years, there have been several studies suggesting that alterations in *SCN5A* gene expression may increase susceptibility to arrhythmogenic diseases, in particular BrS. For instance, Leoni and colleagues reported that mice expressing a single copy of the *SCN5A* gene show cardiac defects that resemble those observed in humans with BrS [116]. However, at present, the molecular mechanisms of *SCN5A* transcriptional regulation are still not well understood. In this regard, our laboratory has provided some insight into these mechanisms, by showing that GATA4 and GATA5 TFs co-occupy the *SCN5A* promoter and the first intronic regions, and synergize in order to regulate the expression of the *SCN5A* gene in the human heart [117]. A recent report has also shown that IRX5 and GATA4 act synergistically to activate the *SCN5A* promoter [118].

Different studies have identified a series of common SNVs in the *SCN5A* core promoter that are associated with alterations in the transcriptional activity of *SCN5A*. Bezzina and colleagues described three haplotypes (HapA–C) within the *SCN5A* promoter region, consisting of different combinations of six common SNVs in near-complete LD (rs41311113, rs9825294, rs41310241, rs41310239, rs41310237, and rs41310236) [102]. Luciferase reporter assays showed that HapB, common among the Japanese population, is associated with a significant decrease of the promoter activity. Interestingly, while the haplotype is present in 22% of the Japanese cohort, it is rare or absent in other populations, suggesting that the effects associated with this haplotype could be population-specific [102]. Another example was described by Yang and colleagues, who described a haplotype in the *SCN5A* promoter consisting of two common variants, rs41310749 and rs41310239 [119]. They observed that patients carrying the haplotype in heterozygosis with a loss-of-function *SCN5A* coding variant showed a more severe BrS phenotype [120], supporting the notion of a modulatory effect of non-coding variants on the severity of disease. 

In the same line of evidence, Yang and colleagues described SNVs within the 5′ UTR, exon 1, and intron 1 of *SCN5A* in a Caucasian BrS cohort. They also observed that these variants lie within TFBS and are affecting the binding of specific TFs. Similarly, a recent study by Yagihara and colleagues identified novel rare variants within the core region of the *SCN5A* promoter in BrS patients from Japanese origin, observing that three of these variants are associated with reduced promoter activity [121]. An interesting example of a *SCN5A* promoter variant is rs1805124, which has been described as a protective genetic modulator. This common variant has been shown to decrease the methylation rate of the promoter, thereby increasing *SCN5A* expression levels. As a result, rs1805124 carriers present ECG patterns with lower severity, enhanced sodium channel activity, and less frequent occurrence of ventricular fibrillation [122]. 

Altogether, these studies support the notion that variants in the *SCN5A* promoter, and especially those affecting TFBSs, can have an impact on *SCN5A* gene expression and ECG parameters, and therefore modulate BrS pathogenesis.

### 5.2. The SCN5A–SCN10A Locus

GWAS conducted on ECG traits and cardiac conduction disorders have highlighted the association of the *SCN5A*–*SCN10A* locus to QRS duration and PR interval, suggesting a relevant role in cardiac conduction and heart function [84,85]. Remarkably, most of the variants identified in these studies are located in non-coding regions. While *SCN5A* variants are well-known to be associated with cardiac arrhythmias, the identification of *SCN10A* as a major risk region for ECG traits was intriguing. *SCN10A* encodes the alpha subunit of the sodium channel Nav1.8, originally described as being expressed in nociceptive sensory neurons and playing an important role in pain perception [123,124,125]. Later studies have shown that Na_V_1.8 contributes to cardiac electrophysiology, but the molecular mechanism is still under debate, especially regarding its expression in cardiomyocytes. A murine model reported that Na_V_1.8 is present in intracardiac neurons, but not in ventricular cardiomyocytes [126]. Other studies have claimed that Na_V_1.8 contributes to the late cardiac sodium current [127,128], and suggested that Na_V_1.8 could be involved in arrhythmogenesis [129]. However, a recent report showed the absence of functional Na_V_1.8 channels in cardiac cells from rabbit and hiPSC-CMs [130].

Bezzina and colleagues provided more evidence for the relevance of the *SCN5A–SCN10A* locus with the only GWAS performed on a BrS cohort, with 312 BrS patients of European ancestry. In their study, they identified three independent common variants (rs10428132, rs9388451, and rs11708996) in three different loci (*SCN5A*, *SCN10A*, and *HEY2*). The strongest association corresponded to rs10428132, a variant located within the intron 14 of the *SCN10A* gene [18]. The second strongest association, rs9388451, was detected downstream of *HEY2*, encoding a cardiac TF that regulates cardiac electric activity, and is possibly involved in the pathogenesis of BrS [18,131]. The third strongest association, rs11708996, was located within an intronic region of *SCN5A*. This variant had already been previously linked to alterations in QRS and PR intervals, and thus to cardiac conduction [99,119]. Bezzina and colleagues showed that these three loci have a large cumulative effect on BrS risk, as patients carrying more than four risk alleles presented a stronger association to BrS compared with patients carrying fewer than two risk alleles. The authors also found that 1.5% of the European population is harboring more than four risk alleles, suggesting that these three common variants act as modulators of disease susceptibility rather than being causal variants [18]. 

Studies performed by Van den Boogaard and colleagues uncovered another possible role of *SCN10A* non-coding variants in conduction disorders. In particular, they showed that the *SCN10A* intronic variant rs6801957, identified in a GWAS associated with cardiac conduction disease, is located in an enhancer region that modulates the expression of *SCN5A* gene. Interestingly, they demonstrated that rs6801957 disrupts the binding of the T-box transcription factors 3 and 5 (TBX3/TBX5) to this *SCN10A* intronic enhancer, resulting in reduced *SCN5A* expression levels. Furthermore, they also showed that this variant is associated with decreased *SCN5A* expression in homozygosis in left ventricle samples [132,133,134]. Man and colleagues have recently reasserted the crucial role of the *SCN5A–SCN10A* locus for the control of the gene activity and topology, through the study of a super enhancer cluster downstream of *SCN5A.* Interestingly, they found that all the interacting regulatory components in the locus are required for proper *SCN5A* expression and normal cardiac conduction. They also suggested that variants affecting the super enhancer cluster may affect the chromatin architecture of the *SCN5A–SCN10A* locus, and consequently, the expression of *SCN5A* [135].

In summary, the previously described studies demonstrate that the intronic region of *SCN10A* contributes to cardiac electrophysiology, and that non-coding variants within this region can be associated with BrS or other ECG traits [133]. However, the molecular basis underlying the effects of common variants in *SCN10A* is still unclear, since the role of Na_V_1.8 in cardiac function and its expression in cardiomyocytes remains controversial [128,136]. In conclusion, future studies should decipher whether the functional effect of these variants is related to dysregulation of *SCN5A* expression through the role of *SCN10A* as an enhancer, the activity of Na_V_1.8 in intracardiac neurons that regulate cardiac electric activity, or both.

### 5.3. Non-Coding RNAs

Over the past decade, miRNAs have been shown to play important roles in heart development and function. From those, miR-1, miR-25, miR-26, miR-29, and miR-328 have been specifically associated with cardiac arrhythmias [137,138,139,140,141]. Functional studies have demonstrated that miR-24, miR-98, miR-106, miR-200, miR-219, and miR-1270 regulate *SCN5A* expression [12,142,143,144], while miRNAs like miR-125 and miR-153 have been described as indirect regulators of *SCN5A* [142].

To date, there are only a few studies that have identified variants affecting the function of miRNAs that target *SCN5A,* and therefore could be associated with BrS. Daimi and colleagues identified two common variants (rs4073796 and rs4073797) within the *SCN5A* 3′ UTR of a Tunisian family with BrS, which is, to date, the major study linking miRNA-related variants with BrS [12]. In particular, they showed that together, rs4073797 and rs4073796 create an extra binding site for miR-1270, leading to downregulation of *SCN5A* expression. The same study identified another common variant in the same family, rs107822, located 36 bp upstream the miRNA-219 precursor sequence and potentially affecting the structure of the mature miRNA and, in consequence, *SCN5A* expression. A similar study by Song and colleagues confirmed these observations by detecting differential expression of mature miR-219 in the presence of rs107822 [145]. 

In regard to lncRNAs, it is important to highlight that regulation of BrS-associated genes *MHY7*, *IRX5*, and *CACNA1C*, has been linked with lncRNAs [146]. In particular, the lncRNA cluster *Myheart* has been associated with the regulation of *MHY7* through the interaction with chromatin remodelling factors, and has been reported to be significantly elevated in patients with acute myocardial infarction [107]. Other lncRNAs have been described as being associated with inherited arrhythmogenic diseases, such as AF [147]. One example is the so-called KCNQ1OT1, an lncRNA that regulates *CACNA1C* expression through the sponging of miR-384, leading to alterations in the electrophysiological parameters [148]. In agreement with this data, KCNQ1OT1 silencing has been associated with a lower incidence of AF. Intriguingly, multiple lncRNAs have been found to be differentially expressed in AF patients, although their target genes and function are still unknown [149,150]. Altogether, these observations suggest that non-coding variants affecting the function of lncRNAs could also explain the molecular basis of cardiac arrhythmias in which the etiology is still unknown, including BrS.

## 6. Conclusions

Almost 30 years since the clinical description of BrS, multiple studies have been carried out in order to elucidate both the genetic basis and the pathophysiology of BrS. Despite the great advances in this field, there is still a knowledge gap that needs to be addressed before reaching a reliable understanding of the inheritance and the significance of BrS-associated variants. 

**Figure 3:**
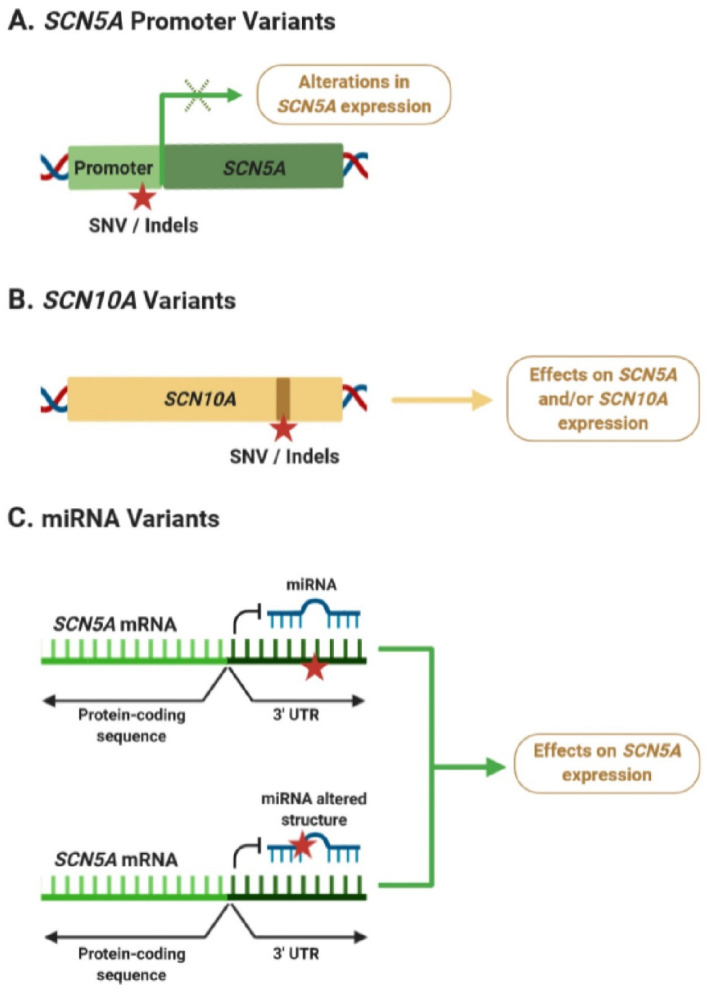
Potential effects of non-coding variants associated with Brugada syndrome (BrS). (**A**) Variants in the *SCN5A* promoter can lead to alterations in its mRNA expression levels. (**B**) Intronic variants in *SCN10A* could be affecting *SCN5A* expression through an enhancer-mediated mechanism or affecting the activity of *SCN10A*. (**C**) Variants within the 3′ untranslated region (UTR) of *SCN5A*, or affecting the function of BrS-related miRNAs, could lead to dysregulation of *SCN5A* expression. Red star indicates the position of a new single nucleotide variant (SNV) or an insertion/deletion (Indel). Green × indicates possible alterations of gene expression.

During the last years, a growing body of evidence suggests that non-coding regulatory variants may play an important role in BrS susceptibility by modulating its phenotype. Many of these studies have identified variants in the *SCN5A*–*SCN10A* locus, which may be affecting *SCN5A* and/or *SCN10A* expression levels in the heart (Figure 3A,B). However, a better understanding of the mechanisms explaining how dysregulation of *SCN5A* and/or *SCN10A* expression is linked to alterations in ECGs and cardiac arrhythmias is still missing. At the same time, only a few studies have focused on examining the potential role of miRNAs in BrS pathogenesis, although their findings strongly suggest that variants within the *SCN5A* 3′ UTR or miRNAs could be associated with alterations in *SCN5A* expression and BrS pathogenesis (Figure 3C).

In this scenario, deciphering the effect of non-coding variants associated with BrS will be critical to further understand the genetic basis of this cardiac disorder, although challenging. The problems encountered by researchers in assessing the role of non-coding variants using hiPSC models are currently difficult to overcome with alternative methods. Murine, porcine, and canine models have been used in the past to study *SCN5A* variants associated with BrS [151]. However, we have to take into account that these models present differences in electrophysiological properties, ion channel expression profiles, or reproductive cycles, and may lead to findings that may not be relevant in humans. Beyond the use of in vitro or in vivo models, we suggest that population studies could be a fine method to better assess the role of common non-coding variants in modulating ECG traits. In this regard, studies of healthy individuals harboring common variants associated with BrS may uncover novel roles of non-coding variants in the regulation of cardiac function.

Even though *SCN5A* is the principal contributing factor to BrS, all the observations aforementioned support the growing hypothesis that BrS pathogenesis follows an oligogenic or multigenic model [15,20,114,115]. Based on this model, we also propose that BrS may not be caused by a single pathogenic variant, but rather by the presence of multiple susceptibility variants acting synergistically through one or more mechanistic pathways. 

In this review, we aimed at pinpointing the increasing evidence suggesting an important role of non-coding variants in modulating BrS susceptibility, and encourage the increase of efforts in the study of non-coding variants, in order to further our understanding of this cardiac disorder.
